# Expression of Concern: Prevalence of type-2 diabetes and prediabetes in Malaysia: A systematic review and meta-analysis

**DOI:** 10.1371/journal.pone.0326888

**Published:** 2025-06-23

**Authors:** The PLOS One Editors

After this article [1] was published, concerns were raised about the search strategy and study selection process used in the systematic review. Specifically:

- The search strategy in S1 Table indicates that the search term “survey*” was excluded, yet community- or population-based surveys are explicitly included in the eligibility criteria.
- The systematic review includes studies reporting repeated national surveys (NHMS-2006, NHMS-2011, NHMS-2015, NHMS-2019; cited as References 28, 32, 35, and 12, respectively, in [1]), which are based on the same population at different timepoints.

These concerns were investigated in consultation with members of the *PLOS One* Editorial Board. The authors were not able to address the concern about the search strategy, and were unable to provide a list of the 786 records identified in the literature search. As a result, the *PLOS One* Editors have concerns about the suitability of the search strategy employed in [1] to address the research question posed, and in addition the article does not comply with the PLOS Data Availability policy.

Editorial Board members advised that only the most recent NHMS ought to have been included in the study, or that if including successive versions of the survey then the authors should have clearly stated their assumptions regarding inclusion of the national surveys and discussed the limitation that individuals may be represented multiple times in the meta-analysis dataset based on their inclusion in multiple iterations of the NHMS.

The consulted Editorial Board members also raised concerns about claims that overreached what was well-supported by the results.

The *PLOS One* Editors issue this Expression of Concern to notify readers of the above concerns, and to relay additional information provided by the authors regarding other errors and omissions in the article:

- There is inadequate justification for including both [2] and [3] in the systematic review, since they appear to have come from the same study sample, and the prevalence rate for [2] may also be artificially low because participants with “known diabetes” were excluded. The authors note that removal of [2] from the meta-analysis for prevalence of type-2 diabetes gives a pooled prevalence estimate of 14.53% (95% confidence interval 12.54–16.65), which is very similar to the original estimate.
- In Table 1, the risk of bias is recorded as “high” for most included studies, but the Results section states “none [were found] to be of high-risk bias”. The authors clarify that the last column heading of Table 1 should be “Methodological Quality”. With this notice, they also supply the completed risk of bias assessments for each study or outcome (S2 Table).
- In the Literature search section of the Methods, “Malaysian Journals Online” refers to local Malaysian journals; an exhaustive list of journals searched was not provided by the authors.
- In Table 1, the National Health and Morbidity Survey 2019 survey should be linked to Reference 12, not Reference 11.

## Supporting information

S2 Table JBI critical appraisal checklist. (DOCX)
