# Supplementary figures and images for: Prevalence of type-2 diabetes and prediabetes in Malaysia: A systematic review and meta-analysis
Source: PLoS One. 2022 Jan 27;17(1):e0263139. doi: 10.1371/journal.pone.0263139 (PMC8794132; doi:10.1371/journal.pone.0263139)

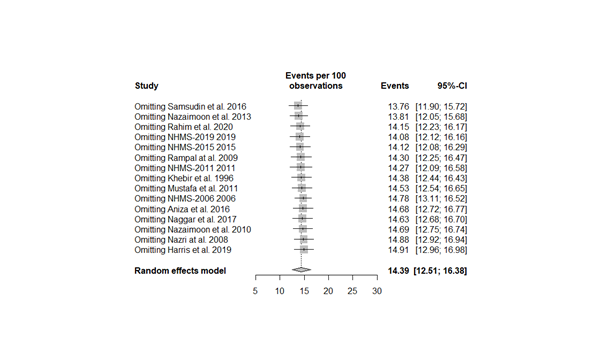

Supplement: S1 Fig — (TIF) [file pone.0263139.s001.tif]

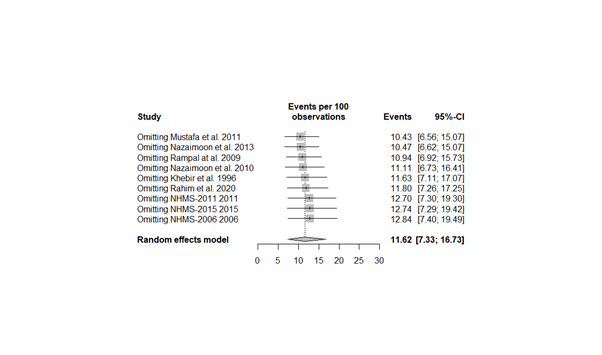

Supplement: S2 Fig — (TIF) [file pone.0263139.s002.tif]
