# Supplementary material for: Prevalence of type-2 diabetes and prediabetes in Malaysia: A systematic review and meta-analysis
Source: PLoS One. 2022 Jan 27;17(1):e0263139. doi: 10.1371/journal.pone.0263139 (PMC8794132; doi:10.1371/journal.pone.0263139)
Supplement: S1 Table — (DOCX) [file pone.0263139.s003.docx]

**S1 Table1**. Search Strategies for Electronic Databases

| **Midline (PubMed) Search** | (Diabetes OR "type-II diabetes" OR "type 2 diabetes" OR prediabetes OR T2D OR "non-communicable diseases" OR "impaired fasting glucose" OR "impaired glucose tolerance" OR "risk factors" OR "risk factor" OR "glucose abnormalities" OR "glucose intolerance") AND (Prevalence OR Epidemiolog*) AND (Observat* OR "cross-sectional" NOT (survey* OR review)) AND (Malaysia* OR Malays)  **Result**= 869 articles  **Searched on** 01/12/2021 |
| --- | --- |
| **EMBASE** | (Diabetes'/exp OR ‘type-II diabetes’ OR ‘type 2 diabetes’ OR prediabetes OR T2D OR ‘non-communicable diseases’ OR ‘impaired fasting glucose’ OR ‘impaired glucose tolerance’ OR ‘risk factors’ OR ‘risk factor’ OR ‘glucose abnormalities’ OR ‘glucose intolerance’) AND (Prevalence OR Epidemiolog*) AND (Observat* OR ‘cross-sectional’ NOT (survey* OR review)) AND (Malaysia* OR Malays)  **Searched** on 01/12/2021  **Result**:1208 articles |
| **Web of Sciences** | (Diabetes OR "type-II diabetes" OR "type 2 diabetes" OR prediabetes OR T2D OR "non-communicable diseases" OR "impaired fasting glucose" OR "impaired glucose tolerance" OR "risk factors" OR "risk factor" OR "glucose abnormalities" OR "glucose intolerance") AND (Prevalence OR Epidemiolog*) AND (Observat* OR "cross-sectional" NOT (survey* OR review)) AND (Malaysia* OR Malays)  **Result**= 359 articles  **Searched on** 01/12/2021 |
| **Manual research** | Google Scholar (147 articles); Malaysian Journals Online **(**99 articles)  Hand searching of reference list of selected articles **(**7 articles) |
