# Supplementary figures and images for: Expression of Concern: Prevalence of type-2 diabetes and prediabetes in Malaysia: A systematic review and meta-analysis
Source: PLoS One. 2025 Jun 23;20(6):e0326888. doi: 10.1371/journal.pone.0326888 (PMC12185020; doi:10.1371/journal.pone.0326888)

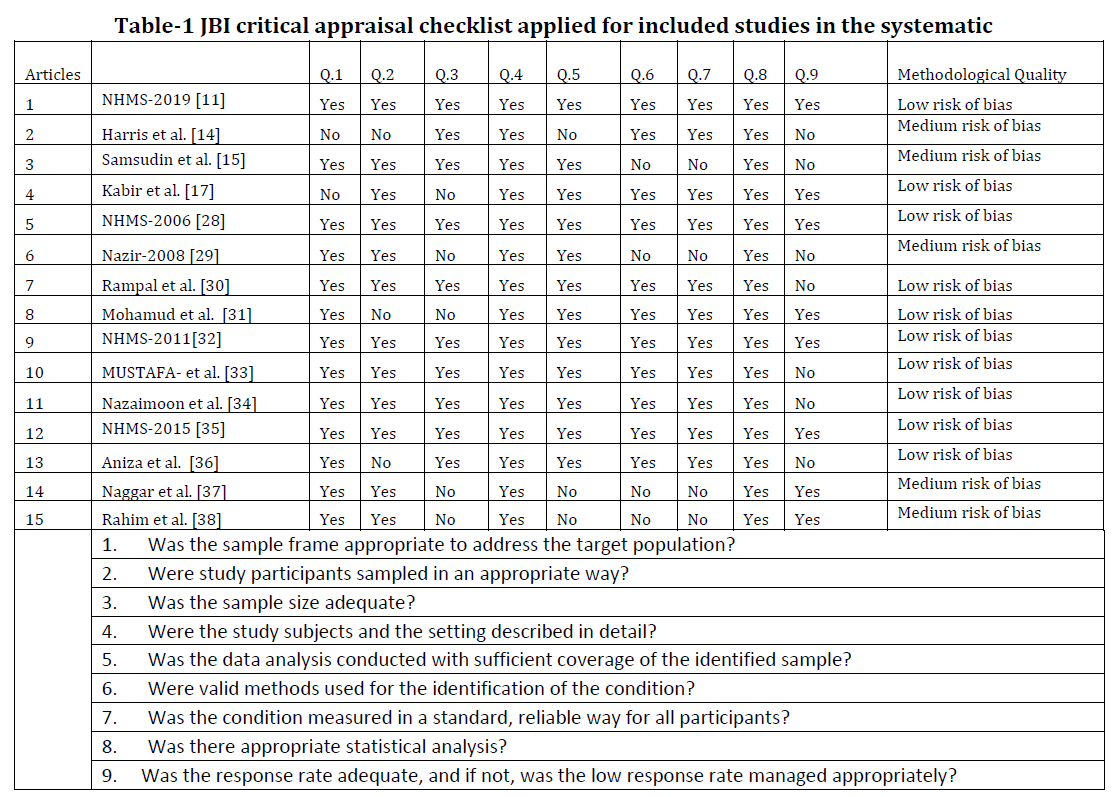

Supplement: S2 Table — (DOCX) [file pone.0326888.s001.docx]
